# Linitis Plastica in a Patient With Previous Breast Cancer: Primary Gastric Cancer or Breast Cancer Metastases?

**DOI:** 10.1002/jgh3.70071

**Published:** 2024-12-20

**Authors:** Ren Yi Jonas Ho, R Rajesh, Wei Keat Wan, Chang Chuen Mark Cheah, Yi Yuan Tan

**Affiliations:** ^1^ Department of Gastroenterology and Hepatology Singapore General Hospital Singapore; ^2^ Department of Anatomical Pathology Singapore General Hospital Singapore

**Keywords:** cancer screening, gastric cancer, linitis plastica, metastases

## Abstract

**Background:**

Linitis plastica is a challenging diagnosis in the setting of a patient with previous breast cancer.

**Case presentation:**

We present a case of a 74‐year‐old Chinese Female with a history of breast cancer who presented with epigastric pain, nausea, early satiety, and weight loss of 5 kg in 3 months. Endoscopic diagnosis of linitis plastica was made, but subsequent management plans were dependent on accurate histological assessment in view of her oncological history.

**Discussion and conclusion:**

Our case highlights the clinical conundrum confronting the endoscopist in evaluating suspected linitis plastica where the imaging, endoscopic, and macroscopic histological findings between primary gastric adenocarcinoma and metastatic breast cancer can be indistinguishable. Optimal deep tissue acquisition is required to differentiate the true diagnosis in such cases.

## 1 Introduction

Linitis plastica is a term with etymological origins whereby “linitis” means inflammatory change, while “plastica” means not pliable. The commonly used moniker of “leather bottle stomach” describes its gross appearance and it is a challenging diagnosis in the setting of a previous oncological history. We hereby present a case of linitis plastica in a patient with a history of breast cancer.

### 1.1 Case Report

We present a case of a 74‐year‐old Chinese female who was admitted under the Gastroenterology service for a 4‐month history of epigastric pain and progressive nausea. Further history revealed a significant unintentional weight loss of 5 kg over the last 3 months with early satiety. Her past medical history was significant for left breast invasive ductal cancer (ER+, PR+, HER+) 4 years prior, for which she underwent a left mastectomy with neoadjuvant radiotherapy and chemotherapy. She was in clinical remission, and maintained on letrozole at the point of admission.

She underwent an esophagogastroduodenoscopy (EGD) which revealed a widely patent and fixed Gastro‐esophageal junction (GEJ). The gastric folds were thickened and prominent with overlying pangastritis associated with severe mucosal edema and sloughing. The stomach was unable to be distended despite copious gas sufflation. Bite‐on‐bite biopsies of the thickened folds were performed with a jumbo forceps with the intent to obtain deep tissue for histologic examination. The duodenum was normal.

Histological examination revealed features of poorly differentiated adenocarcinoma with signet ring cells. Immunohistochemistry demonstrated CDX2 positivity in tumor cells. CK7 and CD20 show variable positivity in tumor cells. In view of her previous history of breast cancer, Staining for GATA3 and ER was performed which were negative. TTF‐1 was also negative. The degree of HER2 overexpression was equivocal. The overall findings were conclusive for a primary gastric adenocarcinoma.

**FIGURE 1:**
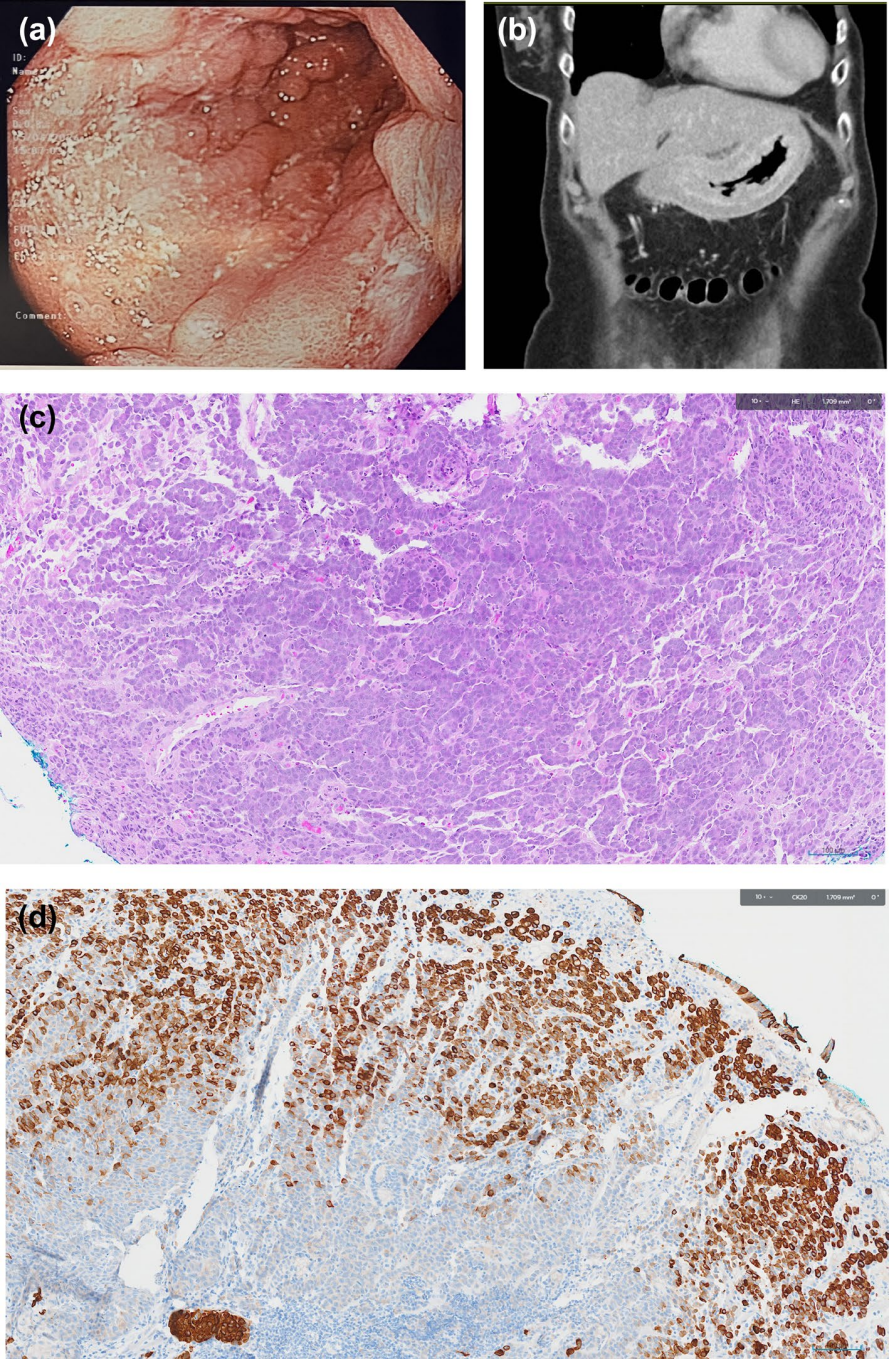
(a) Endoscopic image showing thickened, prominent gastric folds, unable to flatten with insufflation. (b) Computed tomography coronal view showing diffuse mural thickening of the stomach. (c) Histology showing poorly differentiated adenocarcinoma with signet ring cells. (d) Histology showing positive CK20 staining, supportive of gastric adenocarcinoma.

A computed tomography of the thorax, abdomen and pelvis was performed which showed diffuse mural thickening around the distal esophagus and stomach with enlarged perigastric lymph nodes, and peritoneal nodularity (Figure 1). A diagnostic laparoscopy was performed, which confirmed peritoneal metastases from gastric cancer. She was commenced on systemic chemotherapy with a modified FOLFOX‐6 (Folinic acid, fluorouracil, and oxaliplatin) regimen followed by a Paclitaxel based treatment. Unfortunately, her disease progressed and she died 5 months later.

## 2 Discussion

This is an illustrative case of the clinical conundrum confronting the endoscopist in evaluating a suspected linitis plastica in a patient with a background of breast cancer where the imaging, endoscopic and macroscopic histological findings between primary gastric adenocarcinoma and metastatic breast cancer can be indistinguishable. Typical endoscopic findings of a linitis plastica include thickened gastric folds and difficulty in distention on insufflation. Waffle‐like appearance of the upper gastric body due to abnormal folds has also been reported [1]. Linitis plastica is uncommon, accounting for 2%–4.1% of patients with gastric cancer [2]. It has also been known to involve the distal esophagus, with an achalasia‐like presentation [3]. Interestingly, while our patient had radiological evidence of distal esophageal thickening, the GEJ was widely patent and fixed. It is important to note that in a linitis plastica, invasion is often limited to the submucosa and serosal/muscular layers, with superficial biopsies having a yield of only 50% [4]. From the ESMO (European society of medical oncology) 2022 guidelines, locally advanced gastric carcinomas are macroscopically sub‐classified according to the Borrmann classification as polypoid/fungating without ulceration (type I), ulcerated with elevated borders and sharp margins (type II), ulcerated with diffuse infiltration at the base (type III) and diffusely infiltrative with thickening of the wall (type IV)—as in the case of our patient [5]. While the ESMO guidelines recommends 5–8 biopsies for adequate samples to allow for histological and molecular interpretation, it does not elaborate on the ideal location or depth of the biopsy with respect to the subphenotype of gastric cancer. This is a similar shortcoming of the NCCN guidelines (2024). In a Linitis plastica, the depth of biopsy is key to the diagnosis and we recommend the following approach (1) bite‐on‐bite biopsies with a jumbo forceps at index endoscopy. If initial histology is negative, to consider (2) cap‐fitted endoscopic mucosal resection or (3) endoscopic ultrasound‐guided biopsies.

Linitis plastica can result from a primary diffuse gastric malignancy or as metastatic recurrence. Primaries from breast, colon, and bladder cancer have been reported. While more commonly associated with invasive lobular breast cancer, metastatic invasive ductal cancer presenting as linitis plastica can account for up to half of cases in some cohorts [6].

Differentiating primary gastric adenocarcinoma and metastatic breast cancer is of pivotal importance given the downstream implications for treatment and prognosis. Immunohistochemistry remains the definitive means of ascertaining this where positivity for GATA3 and ER and CK7 is consistent in metastatic breast cancer. Conversely, positivity for CK20 and CDX2 supports a diagnosis of gastric adenocarcinoma. This further underscores the importance of optimal deep tissue acquisition to allow for a thorough histological evaluation when evaluating a linitis plastica, especially where metastatic breast disease is a differential.

## Consent

Verbal and written consent was obtained from the patient with regards to publication of their medical information including their endoscopic, radiological, and histological images.

## Conflicts of Interest

The authors declare no conflicts of interest.
